# Analysis of The Working Performance of Large Curvature Prestressed Concrete Box Girder Bridges

**DOI:** 10.3390/ma15155414

**Published:** 2022-08-05

**Authors:** Jian Yuan, Liang Luo, Yuzhou Zheng, Suhui Yu, Jun Shi, Jianan Wang, Jiyang Shen

**Affiliations:** 1 Academy of Combat Support, Rocket Force University of Engineering, Xi’an 710025, China; 2 Key Lab of Structures Dynamic Behavior and Control of the Ministry of Education, Harbin Institute of Technology, Harbin 150090, China; 3 School of Field Engineering, Army Engineering University of PLA, Nanjing 210007, China; 4 School of Civil Engineering, Central South University, Changsha 410075, China

**Keywords:** curved bridge model, mechanical property, numerical shape function, torsion effect, stressing state, box-girder bridge

## Abstract

Based on numerical shape functions and the structural stressing state theory, the mechanical properties of the curved prestressed concrete box girder (CPCBG) bridge model under different loading cases are presented. First, the generalized strain energy density (*GSED*) obtained from the measured strain data is used to represent the stressing state pattern of the structure; then, the stressing state of the concrete section is analyzed by plotting the strain and stress fields of the bridge model. The stressing state pattern and strain fields of the CPCBG are shown to reveal its mechanical properties. In addition, the measured concrete strain data are interpolated by the non-sample point interpolation (NPI) method. The strain and stress fields of the bridge model have been plotted to analyze the stressing state of the concrete cross-section. The internal forces in the concrete sections are calculated by using interpolated strains. Finally, the torsional effects are simulated by measuring the displacements to show the torsional behavior of the cross-section. The analysis and comparison of the internal force and strain fields reveal the common and different mechanical properties of the bridge model. The results of the analysis of the curved bridge model provide a reference for the future rational design of bridge projects.

## 1. Introduction

Curved bridges are becoming increasingly popular due to their reasonable structural performance, aesthetic properties and high torsional stiffness, and are widely used to accommodate more complex routes and tight geometric site constraints [1]. Initially, steel structures were used for curved bridges and later, given the economic practicality, reinforced concrete structures were generally used for small-span curved bridges [2]. Later, the use of prestressing gave the advantages of large spans, large cross-sections, low self-weight, good performance, durability and high torsional stiffness of the box girders. As a result, the application of curved prestressed concrete box girder (CPCBG) bridges has been further expanded in recent years [3,4,5]. However, under the influence of curvature and prestressing, bridges may have complex spatial stress structures and prestressing can have complex effects such as increased torsional effects [6,7].

Although the early analysis of the ultimate load-carrying capacity of curved girders can be dated back to the 1960s [4,6,7,8,9,10,11], regarding the analysis methods, some scholars studied the theory of curved bridges, including pure torsion theory, limiting torsion theory, and bridge type theory, which built the groundwork for the study of CPCBG bridges [12,13]. Subsequently, with the improvement of computing technology, which can realize the operation of a higher-order matrix on the computer, some researchers have used the curve finite band method and finite element method to analyze the deflection deformation and structural force of the prestressed curve bridge [14,15]. However, the analysis method still lags behind the engineering application. Due to the lack of some necessary studies as design references, some functional accidents (such as single pier column urban interchange ramp overturning accident of the main girder due to overload) have occurred [16], prompting scholars to conduct an in-depth study on the necessary working behavior of this type of structure. The following problems of large curvature prestressed concrete box girder bridges deserve critical attention: 1. The complex stressing state of CPCBG bridges, with the presence of a large curvature, makes the bent girders under vertical loads not only subject to bending deformation but also to torsional deformation, with shear stresses, bending normal stresses and warpage stresses in the cross-section existing simultaneously. In addition, second-order effects can also significantly reduce the ultimate load level [17]. The stress level and distribution of CPCBG bridges are related to a variety of factors, such as geometry, the bending and torsional stiffness of the cross-section, support conditions, and loading [18]. In contrast, existing analytical theories and methods find it difficult to achieve an accurate prediction of the structural bearing capacity. 2. The high nonlinearity of the structure and the working behavior involving various parameter uncertainties [19] make CPCBG bridge structures exhibit complex failure mechanisms; the current study of the failure process focuses on the ultimate collapse state [4,8,20,21,22,23], but does not give a clear judgment basis to define when the bridge structure starts to lose it. In fact, this conservative approach is aimed at avoiding and reducing the negative effects of inaccurate predictions, which may lead to considerable material costs and even to the irrationality of the structural design due to the negative impact. At the same time, the degree of reduction in torsional stiffness of the structure and its contribution to lateral deflection-torsional buckling is quite difficult to describe quantitatively due to the development of plasticity. Therefore, it is not easy to find an analytical solution to the relative control equation [20,21].

Hence, a significant amount of research, including model experiments, numerical simulations, analytical theories and methods, have been carried out to perceive the special or invisible characteristics of the working behavior of this type of bridge. Static or dynamic experiments of curved girder bridges have been used to investigate their structural working behavior relative to curvature. For example, YongLi et al. [24] performed dynamic ultimate flexural load capacity tests on prestressed concrete box girders and analyzed scenarios such as the ultimate bending capacity of undamaged and damaged box girders. Kim et al. [25] constructed a 40 m long double girder curved precast prestressed concrete bridge using a multitasking formwork and performed static flexural tests to evaluate its safety and serviceability performance. While numerical simulations of continuous curved box girder bridges were performed to analyze their operational performance considering multiple factors such as span radius of curvature ratio and span length. For example, Khaloo [14] investigated the bending behavior of horizontally curved prestressed box girder bridges using a refined three-dimensional finite element (FE) model and showed that the redistribution of prestressing tendons across the section width can significantly reduce the critical stress. Yang et al. [26] established a numerical model of curved box girder bridges to simulate the tension of prestressing tendons, and then calculated and evaluated the mechanical reflections of bridges with different tension orders to determine the optimal tensile sequence. However, the above experimental data are strain and displacement data of certain measurement points under static or dynamic loading, which only represent the local response of the cross-section, not the whole bridge. Since there are always various errors between numerical simulations and real conditions, it is important to investigate a comprehensive and intuitive approach to study parameters such as strains and internal forces of the whole bridge to further understand the mechanical properties of the bridge.

Structural stressing state theory and non-sample point interpolation (NPI) methods are applied to study the stress characteristics of CPCBG bridges under different loading conditions. Firstly, the characteristics of the structural stressing state embodied in the experimental data of the 1/6 scale bridge model are investigated in depth. The generalized strain energy density (*GSED*) values (*E_ij_*) for different loading cases are used to describe the stressing state of the structure; the strain distribution patterns of the bridge model are plotted to go through the stressing state of the box girder cross-section. Various values of internal forces in the concrete cross-section, specific stress distribution in the cross-section, and torsional effects under different loading cases are clarified. In addition, the experimental data are expanded by interpolation using the NPI method to visually plot the strain and stress fields of the bridge model to analyze the stressing state of the concrete cross-section includes axial force and bending moment. In summary, verified by experimental and simulation data, this study explores a new way to reveal some undiscovered working properties of structures, reveals common and different mechanical properties of bridge models, and provides a new approach to the design and analysis of other types of structures.

## 2. Analysis Method of the Stressing State of Box Girder Model

### 2.1. Retrospective for the Computational Theory of Curved Bridges

Previous analysis theories and calculation methods of curved girder bridges can be summarized into the following three types of analysis: analytical method, semi-analytical method and numerical method. The specific theories are simple torsion theory, warping torsion theory, girder lattice system theory, orthogonal anisotropic plate theory, folding plate theory, polygonal curve theory, internal force transverse distribution theory, finite unit method, finite strip method, energy method and laminated plate method. Among them, the numerical method is the most commonly used. All the above methods have their relative applicability conditions and use characteristics, and the equivalent mechanical treatment of the actual structure is carried out on some assumed mechanical model so that the simulation calculation model can match the structure under actual conditions. Therefore, before applying a certain method, the essential characteristics of the structure are fully grasped, and the corresponding analytical theory and calculation model are used for processing so that the theoretical calculation results can more fully reflect the actual loading case of the bridge structure. Firstly, the pure torsion theory treats the bent bridge structure as an elastic rod concentrated in the center of the beam axis and considers that the cross-section remains flat after loading, i.e., no warpage occurs and the shape of the cross-section remains unchanged, which is based on the pure torsion theory. Second, the warpage torsion theory in the warpage torque in the total torque occupies a considerable proportion. To consider the warpage torsion influence, elastic thin-walled curved rod theory will also be treated as a single thin-walled bending beam for analysis. Thirdly, the combined plate and girder system theory is used for the longitudinal horizontal seam along the intersection of plate and girder to split each main beam rib and deck plate; the deck plate is a multi-span elastic support continuous plate supported on each flexible bending main beam. The deflection of the curved girder and the fan-shaped deck plate is expressed in the form of a sine series and the displacement function is used to express the elastic support reaction force, and the stiffness constant of each plate is derived by substituting the boundary conditions. Then, the differential equation of the curved girder is used to derive the relationship between the external force and the displacement of the beam. Finally, the set of equilibrium equations at the intersection of the plate and the beam is established to solve the displacement function of each bent beam and the internal force is solved. Fourthly, the folding plate theory is to consider the beam as a combination of multiple plates connected, the strain and displacement in the plane of the plate are solved by the plane strain of elastic mechanics, and the internal force and displacement out of the plane are solved by the thin plate theory. Fifth, the principle of the lattice system theory is to replace the bridge superstructure with an equivalent lattice and restore the equivalent results to the original structure to obtain the required calculation results.

Simultaneously, for the finite element analysis method for curved girder bridges, no matter how complex the form of the structure and how many types of loads, the correctly and reasonably established model can be used to analyze and calculate the structure and can analyze the warpage, distortion and shear hysteresis to obtain more comprehensive local stresses as well as the other required stress results. As a result, the simulation of the actual stressing state of the curved bridge is more comprehensive. First, the finite strip method combines the advantages of both the elastic mechanical solution of the orthotropic anisotropic plate and the finite element method as a semi-analytical method that is especially suitable for analyzing regular structures, considering the combined effect of in-plane displacement and out-of-plane buckling of the structure. The displacement function of the finite strip method is usually composed of a Fourier series or spline function in the curve direction and an interpolating polynomial in the radial direction. Then, the finite unit method is solved along the lines of dividing the units, establishing the stiffness matrix of each unit, which can accurately analyze the detailed structure of the bent bridge. However, the time spent is too much and not economical. Third, the plate and shell method, using folded plate approximation or shell unit, correctly establishes the model and carries out a reasonable division of plate and shell units, sets the boundary conditions correctly, and arranges the load according to the actual force condition, so that the displacement and deformation of the structure can be analyzed and various internal forces (torque, bending moment, shear force, axial force) of the bridge structure can also be calculated. Thirdly, the solid method is suitable for local stressing state analysis in some complex parts of the local forces, isolating the analysis object from the whole member. The solid unit method is generally used to establish the critical analysis model and reasonable division of units, and then, based on the relevant boundary conditions, the structure will be discrete in three dimensions. The choice of the solid unit can fully consider the effects of distortion, buckling, shear hysteresis, Poisson’s ratio, etc.

In summary, the analysis of curved girder bridges requires comprehensive consideration of the actual situation of the bridge. Generally, the analysis can be carried out in the following two steps: (i) the beam unit method is chosen for the overall analysis of the structure: (ii) for irregular members or complex force areas. For example, for a large curvature curved bridge, continuous rigid block zero, large volume bearing platform, bull leg, etc., the solid unit or plate and shell unit are selected for further local analysis. All the above methods are based on one or more mechanical assumptions, and although the overall or part of the control, or part of the original analytical model, has been simplified as necessary, the calculation and analysis process is still cumbersome and complicated, and the solution time is long. In addition, there are great difficulties in verifying the results of different methods against each other, not only because the actual mechanical properties still differ greatly, but also the limited and valuable test data cannot be fully utilized. Therefore, the basic method of the structural stressing state theory is adopted here to fully analyze the test data collected, and the NPI method is constructed to expand the test data and reveal some hidden mechanical properties of the bent bridge.

### 2.2. Modeling and Theory of Stressing States of Curved Box Girder Bridges

The concept of structural stressing state is essential in structural analysis and is gradually accepted by most researchers. However, for the complex and continuous structural stressing state during loading, there is no uniform and accurate definition in the corresponding codes so far. Therefore, according to the mutually varying and cross-progressive characteristics presented in the structural working process and the structural behavior revealed by classical mechanics, the structural stressing state can be defined as the internal or external working behavior under certain loading cases, which characterizes the response of different distribution modes and reflects the forms of strain, generalized strain energy density (GESD), displacement, deflection, rotation angle, internal force, etc. at the critical points of the structure. In other words, the stressing state of the structure can be fully represented by a numerical model consisting of the relevant point mechanical responses.

The structural response of box girder bridges subjected to stressing states is evaluated by numerical models to characterize the structural work behavior. Stress and stress data are collected as raw data for experiments and can be used to analyze the deformation characteristics and the working behavior of the structure. Numerical models and corresponding characteristic parameters are constructed in the form of arrays or cells (matrices). A microscopic scalar related to strain and stress [5], i.e., the generalized strain energy density (*GSED*), is used to represent the stressing state at the measured points of the box beam. Due to their experimental error and subjective nature of selection, other structural response parameters (e.g., deflection and crack width) are considered to be limited in reflecting the internal or external performance under loading. The application of the *GSED* parameters presented above can better demonstrate the correct working characteristics of the structure. For the measurement points of the box girder structure, the *GSED* values can be expressed as [27].

(1) $$E_{i}=\int \sigma_{x}d\varepsilon_{x}+\sigma_{y}d\varepsilon_{y}+\sigma_{z}d\varepsilon_{z}$$

where *E_i_* is the *GSED* value of the *i*-th measurement point under the *j*-th loading cases; *σ_x_, σ_y_*, *σ_z_* and *ε_x_*, *ε_y_*, *ε_z_* are the nominal stress and nominal strain in the three orthogonal directions, respectively.

Regarding the concept of strain energy density, in this paper, the generalized (or quasi) strain energy density (*GSED*) is chosen as the characteristic parameter to express the stressing state at the measurement point [28]. Therefore, Equation (1) is simplified as.

(2) $$E_{i}=\frac{1}{2}\sum_{N=1}^{3}E\varepsilon_{N}^{2}$$

where *E_i_* is the *GSED* value at the *i*-th measurement point; *ε_N_* is the nominal strain in the *N*-th direction; and *E* is the modulus of elasticity. The sum of *GSED*s of a group of key points can be calculated by the following Equation (3).

(3) $$E_{sum}=\sum_{i=1}^{n}E_{i}$$

where *E_sum_* is the sum of the *GSED*s of all measurement points of a section of the specimen, representing the stressing state on the entire structure or component (control section, connector, etc.). *n* is the total number of all critical measurement points on the relevant component in the stressing state.

### 2.3. The Method of Non-Sample Point Interpolation

In structural analysis, the limited structural response data collected from experiments can, to a certain extent, reflect the working characteristics of a structure under load. However, it cannot provide the complete expression of the response mechanism and characteristics of the structure. In the experimental part of the stressing state analysis, the strain measurements at several points of the cross-section are used to construct the characteristic values to approximately characterize the stressing state of the cross-section, and this modeling analysis can reflect the changing characteristics of the stressing state of the cross-section to a certain extent, but the stressing state model and physical meaning are not strict enough and not intuitive enough. Therefore, it is necessary to reasonably extend the measured data on the cross-section to obtain more accurate cross-sectional eigenvalues, and on this basis to realize an accurate analysis of the stressing state in the field region of the structure (represented by the cross-section), to reveal the concealed mechanical rules behind the test.

A non-sample point interpolation (NPI) method of response simulation that combines numerical simulation with experimental response samples to improve the interpolation accuracy is introduced here, and a specific response simulation is proposed. The method is based on the concept of shape function in the finite element method to construct discrete weight functions by generalized numerical simulation of a specific ideal model. The numerical shape function is used to adjust the a priori model response results of traditional simulations; the available model information is also considered, which can further improve the interpolation accuracy. The stochastic simulations are then performed by fitting the model with different sample distributions and loadings. The unique structure of the method, with no optional parameters and precise physical meaning, allows for more accurate estimates compared to ordinary simulated values.

Although the assumptions and properties of the numerical shape functions differ, almost all methods use the following estimation formula.

(4) $$u\left(x_{0}\right)=\sum_{i=1}^{m}w_{i}\left(x_{0}\right)u_{i}$$

where *u*(*x*_0_) is the estimated value at any point *x*_0_ after interpolation; *W_i_* is the weighting function assigned to the *i*-th sampling point with observation *u_i_*; and *m* denotes the total number of sampling points. The above is based on the construction of the weighting function.

Additionally, in the finite element, the same Equation (4) is used for the interpolation of the intra-cell displacement. Here, the weight function is denoted as *N_i_* and is referred to as the shape function. The displacement field is represented as a linear combination of shape functions expressed by explicit polynomials. For the calculation of the stiffness equation to converge to a stable solution, the shape function needs to satisfy the following four properties.

1. For the shape function *N_i_*, it has a value of 1 at node *i* and is bounded at all remaining nodes;
2. The continuity of physical quantities between adjacent elements should be guaranteed. It must have at least C0-continuity to form a smooth displacement field;
3. The shape function must contain linear terms to satisfy the constant strain condition. In other words, to make the shape function have a simple mathematical form, priority should be given to the lower polynomial;
4. The shape functions can be linear systems but must satisfy that their sum is equal to the constant one, i.e., *∑N_i_* =1.

Herein, we only try to use the useful properties of shape functions for interpolation, not to improve them for finite element analysis. In general, common simulation functions, including the shape function interpolation method, are versatile and general but have low accuracy for specific problems due to their little physical significance. However, since the calculation of the unit stiffness matrix and the assembly of the global stiffness equations are based on the virtual displacement principle and the force balance principle, respectively, the simulation of a finite element model assembled with a sufficient number of small units can reflect the real response field. According to the concept of discretization and the four important properties mentioned above, the weighting function *W_i_* can be obtained by a numerical simulation of the specific model.

## 3. Experimental Bridge Model

### 3.1. Configuration of CPCBG Bridge

**Figure 1:**
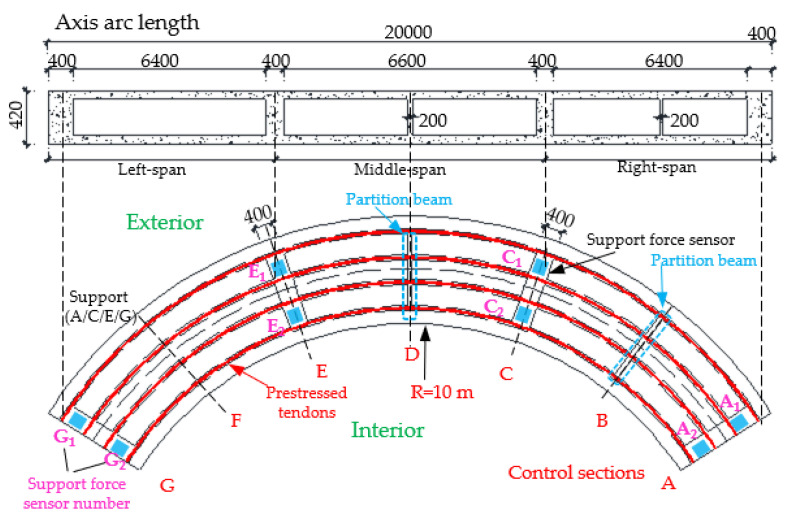
CPCBG model bridge top view and side view (units: mm).

Considering the unique bending-torsional coupling nature of curved girder bridges leading to their complex force characteristics, Shao [29] designed and tested a large curvature prestressed concrete three-span continuous curved box girder bridge model according to the scale of 1:6, to study the actual force characteristics of the curved girder bridge, based on a prototype ramp quadruple bridge for a cross-river bridge project. the above tests satisfy the requirements of geometric similarity, boundary conditions and physical conditions. The bridge model is shown in Figure 1. Seven control sections are set up in the model, namely, supports A and G on both sides, supports C and E in the middle, span B in the right span with spacer, span F in the left span without spacer, and span D in the middle mid-span. The total design axial length and curve radius of the whole bridge are 20 m and 10 m, respectively. Since the test model is a large curvature curved box girder bridge, the significant torsional effect leads to different force characteristics on both sides, so the interior side and exterior side are used to distinguish them. The span thickness of span B and D is 200 mm (marked by blue dashed boxes). To compare the effect of the partition beam on the force characteristics of the bridge, only span F of the left span without the partition beam, and the rest of span B and D are provided with the partition beam. The model of the continuous girder bridge adopts bi-directional support in the middle and fixed hinge support at both ends. The bearing type adopts plate rubber bearing, which can effectively absorb the impact energy and reduce the uneven vertical displacement of the bearing during the loading process by its excellent deformation performance.

**Figure 2:**
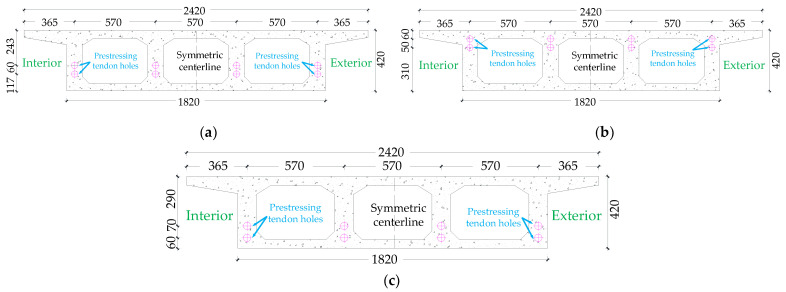
CPCBG model prestressing hole setting sections (units: mm): (**a**) middle span midspan: D control section, (**b**) support: A, C, E, G control section; (**c**) both side span midspan: B, F control section.

**Figure A1:**
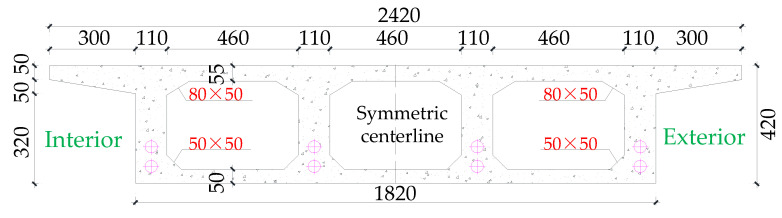
Bridge model typical section size (units: mm).

The cross-sectional dimensions of the box girder are shown in Figure 2 and Figure A1, the thickness of the top and bottom plates of the cross-section are 55 mm and 50 mm, respectively, and the thickness of the web of the cross-section is 110 mm. Since there are obvious differences between the tensile and compressive stressing states of the top and bottom plates at the support and mid-span of the continuous bridge model, and the prestressing tendons are always set in the tension zone, the location of the holes reserved for the prestressing tendons in each control section is different. Figure 2a shows the control section of D in the middle span, Figure 2b shows the control sections of supports A, C, E and G, while Figure 2c shows the control sections of B and F in the middle of the left and right spans.

**Figure 3:**
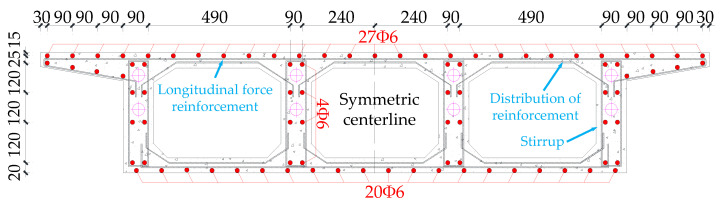
Details of the arrangement of longitudinal reinforcement, distribution reinforcement and stirrups for the bridge model (units: mm).

**Figure A2:**
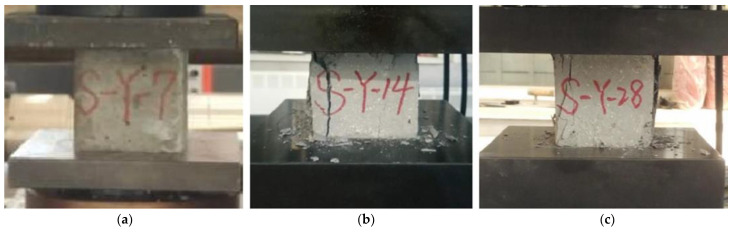
C50 concrete specimens at different ages: (**a**) 7-day compressive strength specimens, (**b**) 14-day compressive strength specimens; (**c**) 28-day compressive strength specimens.

Figure 3 shows the arrangement of reinforcement in the box girder section. Longitudinal reinforcement, hoop reinforcement, distribution reinforcement and prestressing reinforcement are installed in the model box girder. The diameter of common reinforcement is 6 mm, and the diameter of prestressing reinforcement is 15.2 mm. The diameter of the bellows in the web is 18 mm, and the box girder is prestressed and tensioned by the post-tensioning method. The model material concrete is C50 commercial concrete, and all common reinforcement is HRB400 steel. The prestressing tendons are unbonded prestressing strands, and the prestressing tendons are configured as two layers of two rows of Φ15.2 prestressing strands arranged in the web of the girder. The concrete mix ratios are shown in Table 1. the test damage states of the specimens at different ages under standard curing conditions of 7, 14 and 28 days are shown in Figure A2. The compressive strength of the cubes is shown in Table 2, and the average elastic modulus of the specimens is 33 GPa as shown in Table 3. The measured yield stress, elastic modulus and ultimate strength parameters of various steel materials are shown in Table 4.

### 3.2. Control Sectional Measurement Content

**Figure 4:**
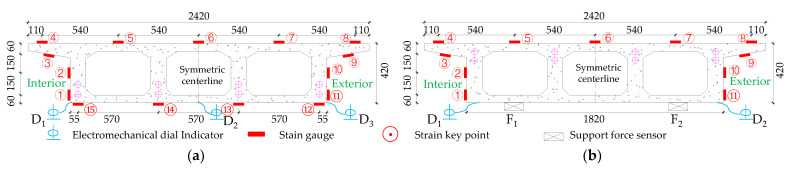
Bridge model section size and detailed instrumentation plan (units: mm): (**a**) Section size information, (**b**) Instrumentation arrangement of midspan section.

The test needs to measure the strain and displacement of the corresponding control section and the bearing reaction force. Firstly, to accurately measure the bearing reaction force, pressure transducers were arranged under the four bearings set up in the whole bridge, as shown in the blue squares in Figure 1 below. The specific positions were left right bearing A and G control section and middle bearing C and E control section, and each bearing measured the inner and outer bearing reaction force (for example, G section bearing, numbered G1 and G2 indicate the outer and inner reaction force, respectively). In addition, the outer and inner deflection displacement values were measured at the span section and the support section of the box girder with electromechanical dial indicators, as shown in Figure 4a, b. Three instruments (D_1_–D_3_) were arranged at three equal points at the bottom of the box girder for the span section, and two instruments (D_1_ and D_2_) were arranged at both edges of the bottom of the box girder for the support section. In the actual test, if the measurement points were at the edges, an auxiliary rod was welded and extended and connected to the micrometer for measurement to prevent dropping. Finally, the strain gauges recorded the test data for the mid-span sections (B, D and F) and the intermediate support sections (C and E). There are 15 strain measurement points (① to ⑮) in the mid-span section and concrete strain measurement points (① to ⑪) in the intermediate support section. The difference between the two is that the strain in the base plate cannot be measured in the support section due to the presence of the support. The specific point arrangement is shown in Figure 4, and the strain gauges of the corresponding measurement points are pasted on the surface of the girder. To achieve the measurement of the above physical quantities, all data records of the test were collected by the Static wired acquisition system. The data sampling frequency is 200 Hz (T = 0.005 s).

### 3.3. Loading Scheme and Test Conditions

**Figure 5:**
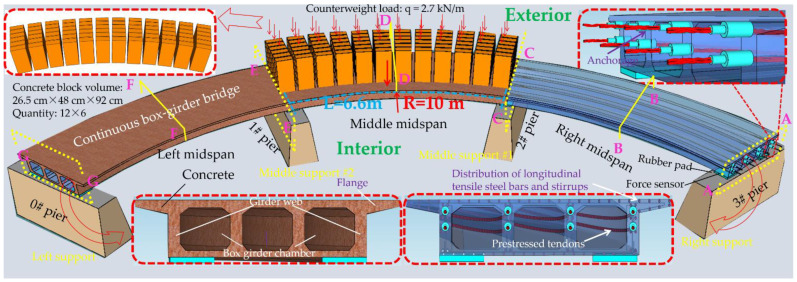
Loading device diagram (Loading case 7: Counterweight load of span C–E). The A–G section represents the key section number of the bridge model.

As shown in Figure 5, the box girder model was loaded by the gravity loading method. First, the prestressing load was applied by tensioning at both ends (post-tensioning method), and the prestressing tendons were unbonded prestressing strands, i.e., no grouting was applied in the bellows in the web. Pre-loading was carried out before the test to bring all parts of the box girder model into the loading case and coordinate with each other. The bridge constant load was applied using a counterweight, using single-span loading and finally effected the superposition. The live load is also loaded utilizing counterweight blocks, and the loading quantity is calculated according to the internal force effect of each control section under the design load, and the load is arranged according to the influence line to achieve the most unfavorable effect of the control section. The loaded counterweight blocks are 2650 mm × 480 mm × 920 mm steel plate concrete blocks, each weighing 300 kg. As shown in the loading schematic diagram of the middle span of uniform loading (loading case 7) in Figure 5, the number of counterweight blocks is 12 × 6, the equivalent uniformly distributed load is 2.7 kN/m, and the details of each section in the loading are given. The loading sequence of the counterweight blocks is from the inside to the outside and from the support to the middle of the span. The loading process is continuous and no excessively long pause is allowed in the middle process. The above test loading setup is mainly to achieve the following purposes.

1. Setting up eight loading cases, as shown in Table 5, to study the effects of the model bridge under constant load, live load and prestressing;
2. To superimpose each constant load condition to obtain the effect of the control section of the whole bridge under constant load.

## 4. Stressing State Analysis of Large Curvature Prestressed Concrete Curved Box Girder Bridge with Different Loading Cases

### 4.1. Investigation into the Sum of GSED (E_sum_) with Different Loading Cases

To reflect the structural stressing state characteristics of the CPCBG curved bridge model, the *GSED* values of the key points on the cross-sections under each loading case were assembled into a matrix representing the stressing state modes

$S_{j}={\left[\begin{array}{ccc} e_{B1} & \ldots & e_{\mathrm{BN}}\\ \ldots & \ldots & \ldots \\ e_{F1} & \ldots & e_{\mathrm{FN}} \end{array}\right]}_{j}$, where *e* is the *GSED* value for each measurement point on the critical sections (B, C, D, E, F and G), where *N* is taken as 15 for B, D and F span sections and 13 for C and E support sections. The parameter *E_j_* is designed to characterize the sum of *GSED* values of the stressing state of the structure control section:

(5) $$E_{j}=\sum_{i=1}^{N}e_{ij}$$

where *e_ij_* is the *GSED* value of the *i*-th critical point at the *j*-th loading case. So far, the structural work behavior modeling based on the structural stressing state analysis theory has derived the stressing state mode *S_j_* and its characteristic parameter *E_j_*. The following study of the changes in *S_j_* and *E_j_* with increasing load will reveal the changing characteristics of the stressing state of the CPCBG curved bridge model.

**Figure 6:**
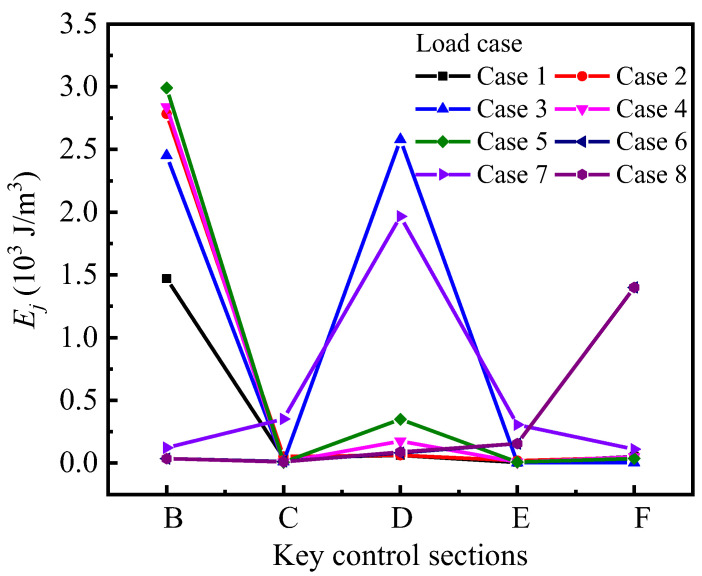
Sum of generalized strain energy density (*GSED*) for each key section with different loading cases.

Hence, the sum of *GSED* values (*E_j_*) of the cross-sections under each loading case can be calculated by Equation (5), and the specific values are shown in Table A2. Then, *E_j_* is plotted with each cross-section-variation curve to reflect the changing characteristics of the structural stressing state. As shown in Figure 6, the magnitude of the sum of *GSED* values of cross-sections reflects the deformation energy of cross-sections, and the span cross-sections (B, D and F) are larger than the support cross-sections (C and E); meanwhile, the *E_j_* values of the span cross-sections in the middle loading section and the left span cross-section are larger in loading case 3, but there are span cross-sections with smaller *E_j_* values due to the presence of the span spacer. The span spacer can effectively reduce the deformation caused by indirect loading. Comparing each loading case, the *E_j_* value corresponding to the loaded section will be larger, while the *E_j_* value of the adjacent section will be smaller because the effect of loading is weakened.

### 4.2. Stressing State Distribution Model

**Figure 7:**
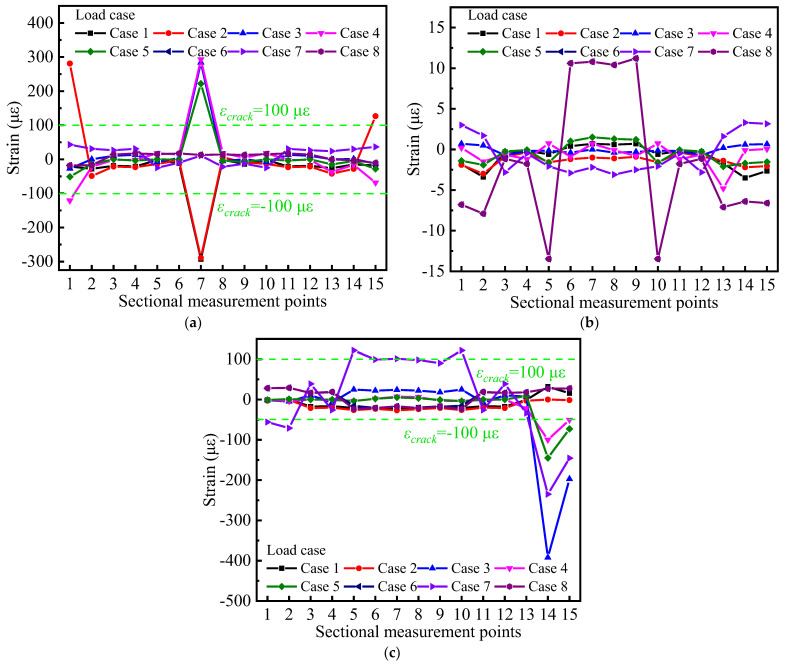
Strain distribution mode of the cross-section with different loading cases: (**a**) distribution mode of section B—partition beam; (**b**) distribution mode of section D no partition beam-partition beam; (**c**) distribution mode of section F-without partition beam.

The strain-based stressing state pattern is plotted as shown in Figure 7. The stressing state mode of each cross-section of the curved bridge model remains slightly variable and stable with different loading cases, with most of the measured points having low strain levels (strain magnitude less than the concrete cracking strain *ε_crack_* = 100 με) and only some of the measured points having large abrupt changes/increments. As shown in Figure 7a,b, the strain distribution mode of the control section with the partition beam is symmetric, but the distribution model of the F control section without partition beam in Figure 7c is asymmetric, especially in the loading case; where the load is applied on the section without partition beam, this asymmetry is more obvious. The main reason for this phenomenon is caused by the torsional effect of the curved bridge model, which also causes this phenomenon at measurement points farther from the symmetry axis of the section. The measurement points farther from the symmetry axis of the section resist a larger overturning moment, resulting in a larger deformation, especially at the bottom edge and web of the box girder.

In general, from the experimental strain data, the bridge model section with a partition beam has a higher load-carrying capacity than the bridge model section without a partition beam, and the strain is more coordinated between deformation and force at the measurement points in each section. Due to the torsional effect of the curved bridge model, the torsional stiffness is smaller than the bending stiffness. Even if the material has the subsequent bearing capacity, the curved bridge model will have local damage under a smaller gravity load, which shows more prominently in the inner side of the curved bridge model. Therefore, in practical engineering, it is important to strengthen the torsional stiffness of curved bridges, such as the reasonable setting of spacer beams. Due to the influence of the large curvature of the curved bridge model, the area around the inner and outer webs of the box girder must be considered vulnerable to damage, and therefore should also be taken into account in the structural design.

### 4.3. Torsional Effects Constructed from Displacements

**Figure 8:**
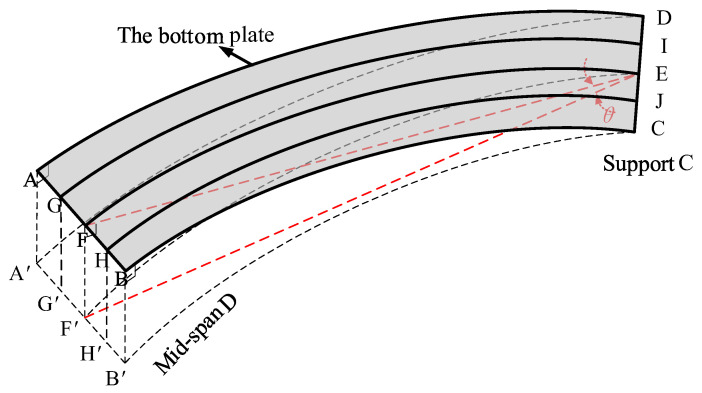
The in-plane bending deflections and rotational angle *θ* for a section.

The curvature of bridge models inevitably brings about differences in vertical deflections and strains at symmetrical external and internal points on the cross-section, which complicates the representation of bridge model mechanical properties in terms of torsional behavior, while the differences in deflections are related to the torsional and out-of-plane behavior of the cross-section, respectively. Therefore, it is desired to understand the effect of curvature on torsional behavior through the torsional effect of displacement construction. The macroscopic expression of in-plane bending moment and torsion is the vertical deformation of the cross-section, so the remaining displacement can roughly represent the direction and degree of torsion of the cross-section by removing the effect of in-plane bending moment on displacement [30]. Since the object of this study is a large curvature box girder, the effect of torsion on the bridge is not negligible, so the measured displacements are used to construct the torsion effect for analysis to verify the trend of the working state with increasing load. For the section deflection at the inner and outer measurement points, the measured displacement can be roughly estimated in bending deformation as shown in Figure 8. Camber ABCD is the base plate of the bridge model from the middle of the span to the bearing. The straight-line CD has zero deflection at the bearing and can be regarded as the rotation axis of camber ABCD. The bending stressing state in the cross-sectional plane can be roughly expressed as the angle of rotation *θ*:

(6) $$\theta =\arctan(\frac{FF^{\prime }}{EF})$$

where *FF’* is the deflection at point F and *EF* is the distance from point E to point F. Therefore, the deflections AA and BB generated by the in-plane bending deformation of camber ABCD at points A and B can be approximated by Equation (7):

(7) $$AA^{\prime }=AD\tan(\theta ),\;BB^{\prime }=BC\tan(\theta )$$

where *θ* is the rotation angle of camber ABCD around support CD; where *AD* is the distance from point A to point D and BC is the distance from point B to point C. Then, the torsional angle *ϕ_i_* of the box girder can be approximated as Equation (8), and the corresponding torsional behavior *T_d_* can be expressed by Equation (9).

(8) $$\phi_{i}=\frac{d_{Ai}-AA^{\prime }}{AF}\;\mathrm{or}\;\phi_{i}=\frac{d_{Bi}-BB^{\prime }}{BF}$$

(9) $$T_{d}=\left(d_{Ai}-AA^{\prime }\right)-\left(d_{Bi}-BB^{\prime }\right)$$

where *d_Ai_* and *d_Bi_* are the deflections at points A and B under the *i*-th loading case, respectively.

**Figure 9:**
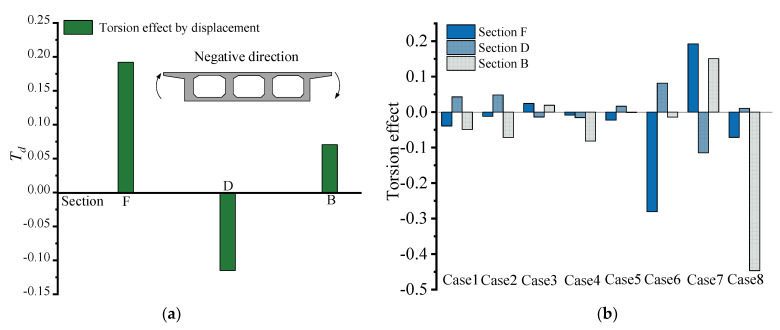
Torsional effect analysis: (**a**) the torsion effect for sections F, D and B for loading case 7; (**b**) The torsion.

Figure 9a shows the torsional effect of the three mid-span sections under the condition of loading case 7, which is specified as negative in the clockwise direction. Under the influence of a large curvature, the displacement on the outside of section D is always larger than that on the inside, resulting in a negative torsional effect under load, and the two adjacent sections F and B have a positive torsional direction. By comparing section B and section F, it can be obtained that the torsional effect of section F is larger than that of section B. This is due to the existence of the cross-sectional spacer in section B. The presence of the cross-sectional spacer will affect the stressing state of the control section to a larger extent. Figure 9b shows the torsional effect of the span section with different loading cases. The torsional behavior of the mid-span section is similar in similar forms of loading cases, such as loading cases 1, 2 and 3, 7 and 4 and 8, 5 and 6. In the mid-span loading case, the torsional direction of the mid-span section is opposite to the torsional direction of the span loading on both sides. Loading on the middle span makes the direction of torsion of the D section negative, while E and F sections are positive (cases 3 and 7); loading on both side spans makes the D section positive, while E and F sections are negative (cases 1 and 2). In loading case 4, under the superposition of the effects of adjacent loads and own loads, the twisting directions of both D and F sections are shown as counterclockwise, and in loading case 5, D and B sections are shown as clockwise and counterclockwise, respectively. When the load is applied at the middle span, the torsional degree of the B section with the cross-section is smaller than the torsional effect of the symmetric F section (cases 3 and 7). On the contrary, the torsional effect of the B-section is larger if the load is applied at both side spans (cases 1, 2; 4, 5; 6, 8).

**Figure 10:**
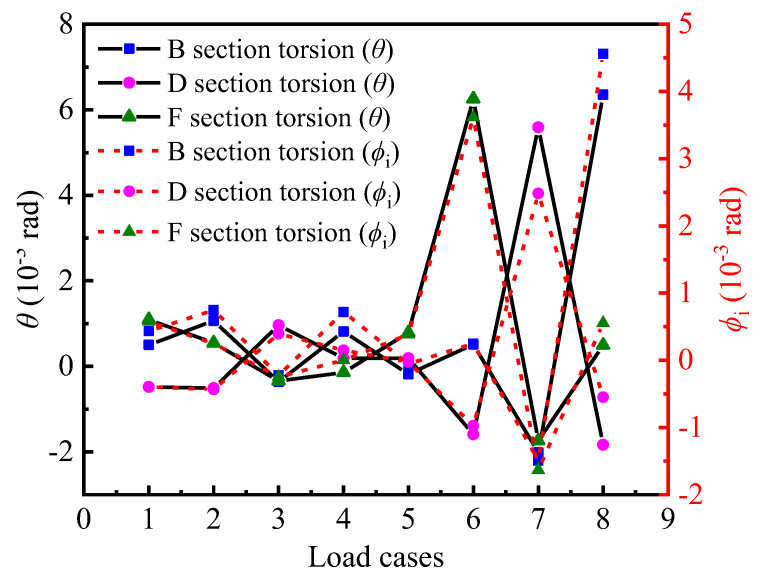
Comparison curves of *θ* and *ϕ* for key sections with different loading cases.

Figure 10 shows the comparison curves of *θ* and *ϕ_i_* at different loading cases, and they reflect the trend changes in the in-plane bending and torsional stressing states of the control section. It can be seen that the trend changes in torsional and bending stressing states at different loading cases are basically the same, and the in-plane bending and torsional stressing states increase sharply with the increase in gravity-loaded test blocks at subsequent loading cases.

The torsional effect modeled by vertical displacement can visually present the torsional distribution, torsional direction and relative magnitude of the torsional effect in the mid-span section with different loading cases. Therefore, by comparing the torsional effects, we can find the common torsional characteristics with different loading cases and better understand the torsional behavior of large curvature box girder models.

### 4.4. Correlation Characteristics between Torsional and Out-of-Plane Bending Stressing States

The bridge models for its large curvature, resulting in its force characteristics, have the following general law: the coupling effect of bending and torsion, bending deformation and torsional deformation superimposed on each other, the same span of bending girder bridge deformation is significantly larger than the straight bridge deformation and, in general, the external deflection is larger than the internal deflection. In the meantime, the interior and exterior girders are not uniformly stressed, resulting in the situation of “unloading of the interior girders and overloading of the exterior girders”. It inevitably produces differences in vertical deflection and strain at the symmetrical interior and exterior points on the cross-section. The differences in deflection and strain are related to the torsional and out-of-plane stressing states in the cross-section, respectively. It is therefore expected to understand the effect of curvature on the torsional and out-of-plane bending behavior; therefore, the parameters *T* and *M_out_* are set to characterize this behavior. Based on the structural stressing state theory, *T* and *M_out_* are characteristic parameters of the torsional and out-of-plane bending stressing states of the cross-section, which can be roughly expressed as Equations (10) and (11).

(10) $$M_{\mathrm{out}\;}=\varepsilon_{\mathrm{out}\;}^{d}-\varepsilon_{\mathrm{in}\;}^{d}$$

(11) $$T=d_{\mathrm{out}\;}-d_{\mathrm{in}\;}$$

where *d_out_* and *d_in_* are the external and internal deflections of the measured section, respectively, and *ε_out_* and *ε_in_* are the external and internal strains of the base plate, respectively.

**Figure 11:**
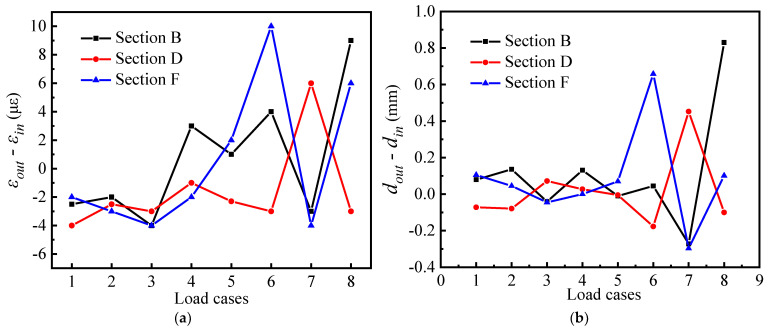
The trend changes of torsion and out-plane bending behaviors for sections B, D and F: (**a**) out-of-plane bending mode; (**b**) Torsional mode.

From the curves of *M_out_* and *T* in Figure 11, the relevant characteristics of the torsional and out-of-plane bending behaviors of B, D, and F sections can be seen: (1) The characteristic parameter T is expressed by the section displacement and can be regarded as the extrinsic expression of the torsional stressing state. The characteristic parameter *M_out_* is expressed by the strain, which can be regarded as the intrinsic expression of the bending stressing state. The change in cross-sectional stressing state can be reflected in the internal manifestation of torsional behavior, i.e., the changing trend of the *M_out_* and *T* curve in torsion and out-of-plane bending is generally consistent with different loading cases. (2) In the meantime, the difference between B and F sections with and without a partition beam can be seen again in Figure 11 for loading case 6, which results in significantly greater torsion and out-of-plane bending in the F section compared to B. The stressing state of the F section is crucial in the mechanical performance of the whole bridge model, since it is the weakest part of the bridge and the main role of the partition beam in a curved girder bridge is to maintain the stability of the whole bridge. Especially, for thin web box girders, the addition of a spacer is the best way to reduce the deformation of the section.

## 5. Experimental Data Extended by the Non-Sample Point Interpolation Method

### 5.1. The NPI Method Applied to CPCBG Bridge Model

Since the measurement points in the experiment can only reflect limited working characteristics and the test cost is high, the experimental data cannot fully reflect the structural response mechanism and working performance of the box girder. Simultaneously, the estimation accuracy of existing simulations is relatively poor, especially for the interpolation of experimental response samples. The general simulations only focus on the spatial distribution and numerical values of the samples without paying attention to their hidden information [31]. Therefore, to overcome the above-mentioned drawbacks of traditional experiments, the new interpolation method with high accuracy and clear physical meaning proposed in Section 2.3 is applied here to be able to make full use of the sample information, especially the complete information of the response data sampled in the experimental model. In addition, accurate simulations with clear physical meaning and reasonable consideration of all available model information can meet the accuracy requirements for in-depth experimental analysis [28]. The extended experimental data obtained through reliable simulations are useful for studying the stressing state of the experimental structure and revealing some global operational characteristics. Therefore, the non-sample point interpolation (NPI) method with explicit physical meaning is proposed to overcome the above drawbacks [31].

**Figure 12:**
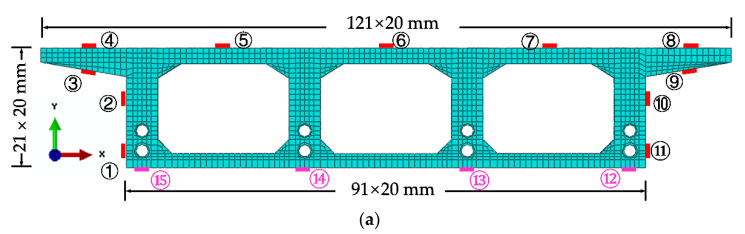

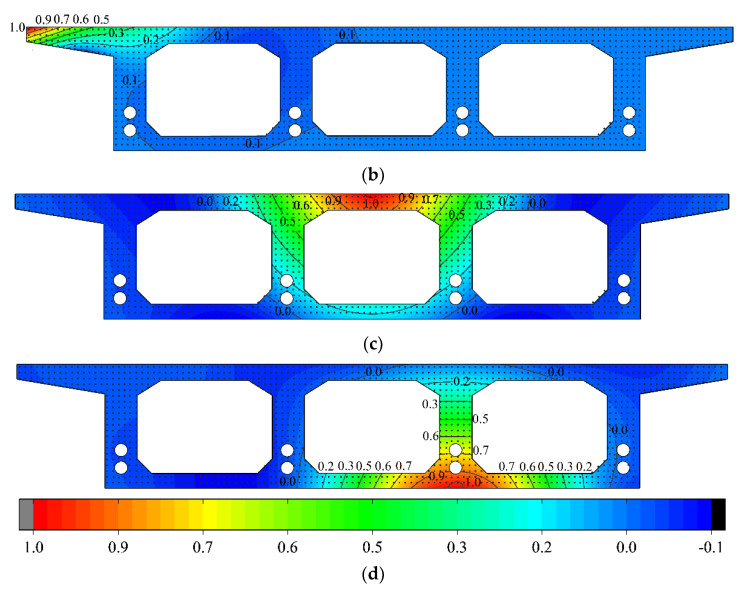
The finite element model of control section and interpolated shape function contour lines map: (**a**) Finite element model and 1–15 control points; (**b**) Interpolation function *N*_4_; (**c**) Interpolation function *N*_6_; (**d**) Interpolation function *N*_13_.

1. The NPI is a new and effective interpolation method that directly interpolates the experimental data field, utilizing conventional shape functions and finite element (FE) simulations. The interpolation method based on thin-plate splines (TPS) obtains the numerical shape function of the sampled points by finite element simulation of a specific thin plate model. Then, the data of non-sampled points are obtained by interpolation of the numerical shape function and the sampled data, and the NPI is constructed as follows. First, to introduce this method vividly, the D cross-section of the experimental model is used as an example. As shown in Figure 12a, ANSYS software is used to build the finite element model of the box girder cross-section, and shell unit 181 is used for the concrete slab with a thickness of 5 mm and a unit area of 10 × 10 mm^2^. Beam element 188 is used to model the common and prestressed reinforcement in the cross-section. Its thickness is also 5 mm, and its area is the actual area. Additionally, it is assumed that the connection between the reinforcement and concrete is rigid, and the prestressing reinforcement is pre-tensioned in advance. The strain data of 15 critical measurement points on the cross-section are used as a sample interpolation to predict the strain field of the critical section in span D.
2. As shown in Figure 12a, the non-sample point shape functions *N_1_-N_15_* are obtained from the 15 control points in the established coordinate system, and the measured point strain data are used as the coefficients of control points ①–⑮. The calculation of the interpolation function can be found in the literature [2,28,32].
3. The 15 interpolation points on the box girder section were used to obtain the z-axis deflection *U_z_*. To interpolate the *U_z_* response field, a shape function can be derived from a generalized finite element simulation of the same physical quantities. *U_z_* = 1 is applied at one measurement point on the z-axis and minimum constrained rigid body displacements in the *x* and *y* directions are applied at the other measurement points. Then, the simulated *U_z_* field is solved by a static finite element method to obtain the shape function for a given measurement point. It can be described by a discrete vector $N_{1}=\left\{N_{1}\left(x_{1}\right),N_{1}\left(x_{2}\right),\ldots ,N_{1}\left(x_{k}\right),\ldots ,N_{1}\left(x_{n}\right)\right\}$, where *N*_1_(*x_k_*) is the value of the element node *x_k_* as a function of the total number of element nodes (*n* = 1612) of the box girder model studied in this paper, and the numerical shape function *N_i_* (*i* = 4, 6, 13) is shown in Figure 12b–d.
4. Static analysis is performed to obtain the overall strain field. Without considering large deformation or elastoplasticity, the displacement field constructed by Castigliano’s theorem is independent of the loading path and can be linearly superimposed according to the simulation results, which have a clear physical meaning.

The above method is used to extend the limited experimental data of the box girder model, which overcomes the shortcomings of the traditional finite element simulation that simply validates the structural analysis and cannot effectively use the experimentally collected data. The numerical interpolation function is obtained from the numerical simulation of a specific physical model. The strain field of the model section is expressed as a linear combination of partial coefficients (measured point strain data) and interpolation functions (displacement field), which can be expressed as.

(12) $$D=\sum_{i=1}^{m}u_{i}N_{i},N_{i}=\left[N_{i}\left(x_{1},y_{1}\right),N_{i}\left(x_{2},y_{2}\right),\cdots N_{i}\left(x_{j},y_{j}\right),\cdots N_{i}\left(x_{n},y_{n}\right)\right]$$

where **D** is the strain field of the entire cross-section, m is the number of strain measurement points (*m* = 15 in this case), *N_i_* is the interpolation function obtained based on the *i*-th control point, *N_i_* (*x_j_*, *y_j_*) is the value of the function of element nodes (*x_j_*, *y_j_*), and *n* is the total number of element nodes (*n* = 1612).

Therefore, the NPI method applied here can be used to extend the limited experimentally measured strains on the cross-section to realize the strain field and internal force distribution patterns of the structure. This method can meet the requirements of in-depth experimental analysis and reveal the overall/local working characteristics of the structure.

### 5.2. Comparison of the Stress Distribution Curves Obtained by the Two Methods

**Figure 13:**
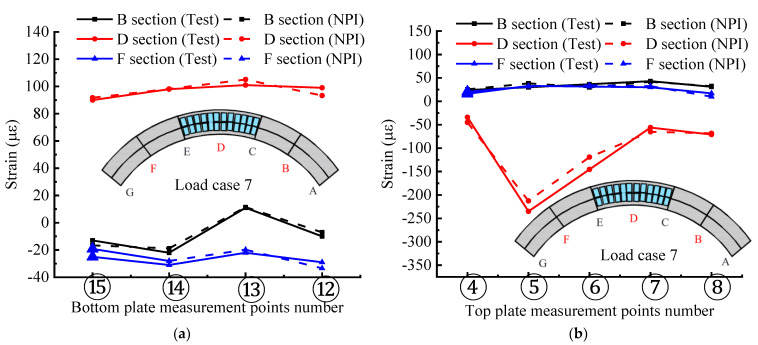

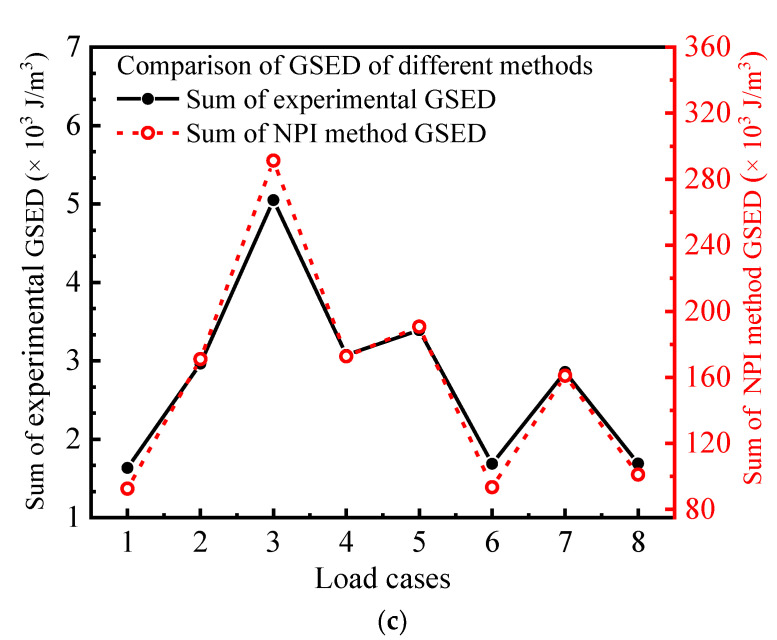
Comparison curve of strain distribution modes of test data and NPI method: (**a**) Strain distribution mode of bottom plate measurement point; (**b**) Strain distribution mode of the top plate measurement point; (**c**) Comparison of *GSED* sums for different methods. The A–G section represents the key section number of the bridge model.

Before applying the NPI method for interpolating stress/strain fields with constructing internal forces, it is necessary to evaluate the accuracy and precision of the NPI method. Herein, the strain data at different locations expanded by the NPI method are used for analysis. The comparison between the NPI method and the experimentally measured strains is given in Figure 13a,b. Here, the comparison of the transverse distribution of strains in the top and bottom plates for different spanwise sections (B, D, and F sections) is indicated. In the case of condition 7, the strain distribution of the bottom plate of the mid-span section is more uniform, and the strains measured by the NPI interpolation method and the test are more consistent, which proves that the NPI method is still very effective in interpolating the strain field.

The above verifies that the strain study of each location of the box girder model can be effectively analyzed by using the strain data unfolded by the NPI method. Additionally, here, the corresponding stress values are calculated by the constitutive relationship of the materials. Thus, the sum of the *GSED* values of the NPI interpolated cross-sections can be expressed by the following Equations (13) and (14) [33].

(13) $$e_{ij}=\int_{0}^{\varepsilon_{j}}\sigma_{i}d\varepsilon_{i}$$

(14) $$E_{sum}=\sum_{i=1}^{N}e_{ij}A_{i}$$

where *e_ij_* is the *GSED* value of the *i*-th element in the *j*-th loading case; *σ_i_* is the normal stress of the *i*-th element; *ε_i_* is the normal strain of the *i*-th element; *E_sum_* is the sum of *GSED* of all control sections in the *j*-th loading case by the NPI method; *N* is the total number of elements; *A_i_* is the area of the *i*-th element.

In Figure 13c, for comparison, the *E_sum_* of experimental strain data is based on Equations (2) and (3) to calculate the sum of experimental *GSED*s, while the *E_sum_* based on the NPI method is used to calculate the sum of *GSED*s of the whole interpolated cross-section, using Equations (13) and (14) for comparative analysis of *E_sum_* variation trends with different loading cases. Therefore, the proposed NPI method is applied with the experimental strain-based method to calculate the *E_sum_* variation curves for different operating conditions for comparison. The ones in Figure 13c reflect similar variation trends and characteristics. (1) The variation trends of the summed *GSED* curves obtained by the two methods are consistent. (2) The *GSED* sum curves of the box girder model show different structural deformation energies in different loading cases, and both methods reflect the maximum structural deformation energy in loading case 3, which can be understood as belonging to the most unfavorable loading mode of the case. (3) Both sets of curves show that the sum of *GSED* (energy curve) can reflect the structural deformation state as a whole based on a limited number of key measurement points with different loading cases. Therefore, this proves that the structural stressing state analysis based on the NPI method can reveal the working characteristics of a curved box girder.

### 5.3. Evaluation of the Comprehensive Accuracy of the NPI Method

The above validation provides a comparative study of the interpolation accuracy of strains at different locations of the box girder model. Here, the overall validation is performed from two aspects.

**Figure 14:**
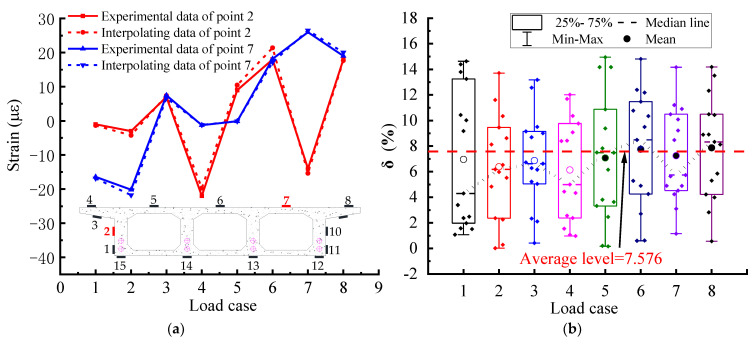
Strain data error results of the Middle midspan D section for CPCBG bridge model with different loading cases: (**a**) Comparison curve between test data and NPI method for points 2 and 7; (**b**) Error of cross-checking for all measuring points.

The first aspect is the strain validation of the same measurement points with different loading cases, as shown in Figure 14a, which validates two measurement points (points 2 and 7) of the mid-span section (section D) of the box girder. The idea of validation is to construct the strain field of the mid-span section with the remaining 13 measurement points (excluding the two points to be validated), obtain the interpolated strain data of the two points to be validated, and then compare the interpolated strain with the test strain of the measurement points. The error δ_ij_ at the *j*-th loading case and the average error of the key point δ can be calculated by Equations (15) and (16).

(15) $$\delta_{ij}=|\frac{\varepsilon_{ij}^{p}-\varepsilon_{ij}^{e}}{\varepsilon_{ij}^{e}}|\times 100\%$$

(16) $$\bar{\delta }=\frac{1}{N}\sum_{i}^{N}\delta_{ij}$$

where, *δ_ij_* is the error between the interpolated and experimental results at the *i*-th measurement point in the *j*-th loading case, and $\varepsilon_{ij}^{e}$ and $\varepsilon_{ij}^{p}$ is the experimental strain and interpolated strain at the *i*-th point in the *j*-th loading case, respectively. *n* is the total number of strain measurement points, and here *N* = 15.

As shown in Figure 14a, the experimental strain curve and the interpolated strain curve vary with different loading cases, indicating that the two curves agree satisfactorily and even partially overlap each other in different loading cases. Additionally, in loading case 6, the two curves start to diverge slightly, but the difference between them is also small and within an acceptable range.

The second aspect is cross-validation. Here, the leave-one-out (LOO) cross-validation method [34] was used, i.e., in the *j*-th loading case (j = 1–8), all the test strain data of the measurement points excluding the *i*-th measurement point to be validated were used to perform interpolated strain, i.e., the NPI method was used to construct the cross-sectional strain field, and the error analysis was performed using the interpolated data of the *i*-th measurement point to be validated, and the test data. Figure 14b shows the validation errors of all 15 measurement points with different loading cases. The overall error ranges from 0% to 15%, with an average error of 7.576%. The error results are relatively small and can fully meet the needs of engineering applications. Meanwhile, the error results are relatively large in loading cases 6 and 8. Due to the small strains at some control section measurement points, the small differences between $\varepsilon_{ij}^{e}$ and $\varepsilon_{ij}^{p}$ are amplified by the too-small denominator in Equation (15), i.e., the error analysis is more sensitive when the strains are small. In general, the average value of the error of the D control section is relatively small for each loading case.

The above analysis and comparison fully satisfy the application requirements and the NPI method can be used to extend the experimental data accurately enough to go deeper into the potential characteristics of the stressing state of the box girder model.

### 5.4. Strain and Stress Fields Extended by the NPI Method

To properly evaluate the stressing state of the CPCBG, firstly, the strains in the non-measured region of the cross-section were calculated by interpolating the limited experimental strain data to obtain the strain field of the control section and the stress field based on the intrinsic relationship. Herein, the above-mentioned NPI principle is used for programming modeling calculations. Finally, the calculated contour maps of the strain and stress fields of the mid-span section are plotted.

**Figure 15:**
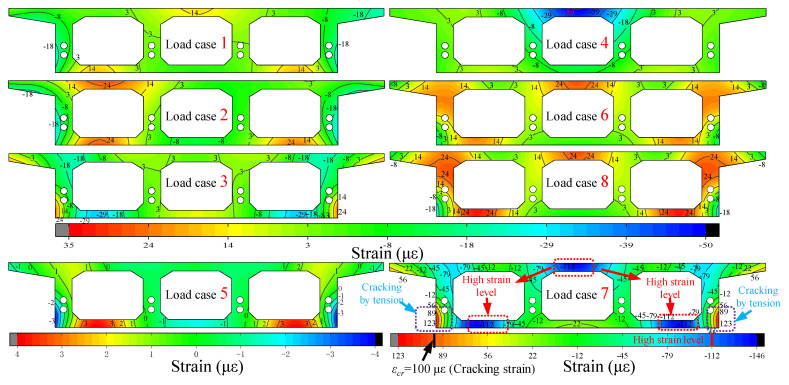
Stress field of the cross-section in midspan D with different loading cases.

For the strain field, as shown in Figure 15, a set of diagrams is used to represent the variation law of the stressing state for different loading cases, all of which indicate the strain state of the D control section in the middle of the span, which visually describes the various characteristics of the stressing state of the box girder structure. The symbols of each group of diagrams are specified as positive for tension and negative for compression. The strain values of the control sections under loading cases 1, 2, 3, 4, 6 and 8 are in the same range interval, and the values of the contour diagrams of the uniform control sections determined by the color cards are equidistant. In general, the blue and red areas in the middle of the concrete top and bottom slabs are varied for different loading cases because the span D control section is subjected to tension on the upper or lower side with different loading cases, which also follow the principle of mechanical influence lines. For example, the blue and red areas of the span D control section in loading cases 5 and 8 are in the opposite situation. In addition, in loading case 7, the tensile and compressive strains change sharply at the intersection of the bottom flange and web on both sides of the box girder. The outermost side reaches the cracking strain, which is due to the large curvature of the box girder structure, and the torsional deformation is more likely to lead to the failure of the bottom two sides of the box girder, which also intuitively reveals the mechanical response of the three-box chamber box girder to the torsional problem. This also indicates that the local location of the box girder is in an unstable state of stress, and therefore is a potential risk, so this part should be given enough attention and reinforced appropriately during investigations.

**Figure 16:**
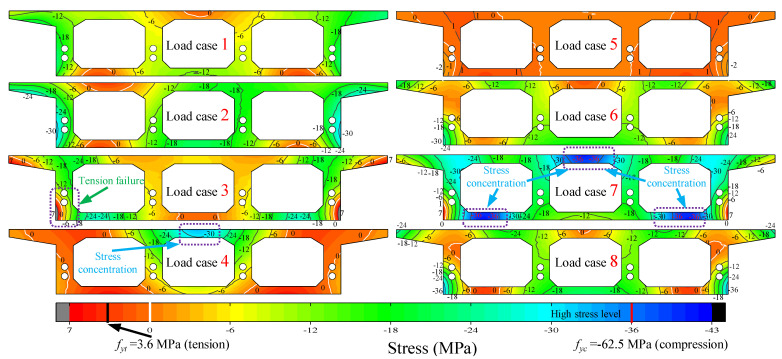
Strain field of the cross-section in midspan D with different loading cases.

For the stress field, in Figure 16, the contours are marked by lines of different colors indicating 3.6 MPa (black), 0 (white), and −62.5 MPa (red), and the corresponding values represent the nominal ultimate tensile stress, the location of the neutral axis, and the ultimate compressive stress of the concrete, respectively. On the whole, the stress contours behave more uniformly under various loading cases, and the stress contours only become dense at local locations, which also indicates that the three-box chamber box girder, as a reasonable structural section form, can effectively involve all parts in coordinating the stresses. In loading case 7, there is also an obvious twisting problem in the middle of the top plate and both sides of the bottom plate in the strain field, which shows the stress concentration. In the case of loading case 6 and loading case 7, the tensile and compressive positions are just opposite, which is the different torsional direction of the span D control section in the CPCBG model under the two different loading cases; the span D control section in loading case 6 is twisted to the outside, while the span D control section in loading case 7 is twisted to the inside. The stress field in case 5 is smaller in Figure 16 because the effect of the left GE span and the middle EC span is offset due to the principle of the mechanical influence line.

**Figure 17:**
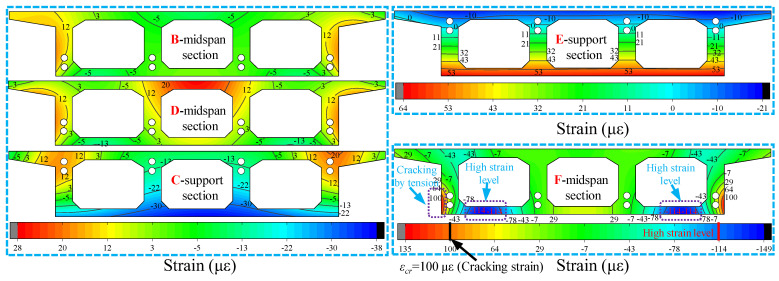
Strain field for different cross-sections with loading case 6.

Meanwhile, to compare the change characteristics of the working performance of different sections of the box girder under the same loading case, Figure 17 depicts the change in strain field before and after different control sections in the case loading case 6. Firstly, the contours of the strain fields of the support C and E sections are close to horizontal, as the torsional deformation of the box girder support section is limited by the support, showing that the box girder support section is still in a horizontal state, while the tensile and compressive tension of the top and bottom plates of the support C and E sections are just opposite, the E section is close to the loading position, and its stressing state is also far from the stressing state of the C section. For the span B, D and F sections, the contour of the strain field is no longer horizontal, and the overall strain on both sides is greater than the strain in the middle, which shows the torsional effect. With the F section as the control section in case of loading case 6, the bottom flange of the box girder and the web intersection are still the location of the sudden change in stress, which also shows the resistance to the maximum torsional effect here and should be given enough attention.

### 5.5. Modeling of Axial Forces and Bending Stressing States

After the interpolated strains of the entire control section are obtained by the NPI method, the internal forces of the control section can also be obtained using the integration principle and the physical meaning of the internal forces. A characteristic parameter with obvious physical meaning and an accurate description of the stressing state is constructed here. Equations (17)–(19) calculate the axial force (*N_j_*), in-plane bending moment ($M_{j}^{in}$) and out-of-plane bending moment ($M_{j}^{out}$) of the control section.

(17) $$N_{j}=\int_{A}\sigma dA=\sum_{A}\sigma_{i}A_{i}$$

(18) $$M_{j}^{\mathrm{in}}=\int_{A}\sigma y\;dA=\sum_{A}\sigma_{i}y_{i}A_{i}$$

(19) $$M_{j}^{\mathrm{out}}=\int_{A}\sigma x\;dA=\sum_{A}\sigma_{i}x_{i}A_{i}$$

where j is the *j*-th loading case, *i* is the *i*-th node within the control section, *σ_i_* is the normal nominal stress at the *i*-th node, *Ai* is the area of the *i*-th node, and *x_i_*, *y_i_* are the horizontal and vertical distances from the *n*-th node to the neutral axis of the section.

**Figure 18:**
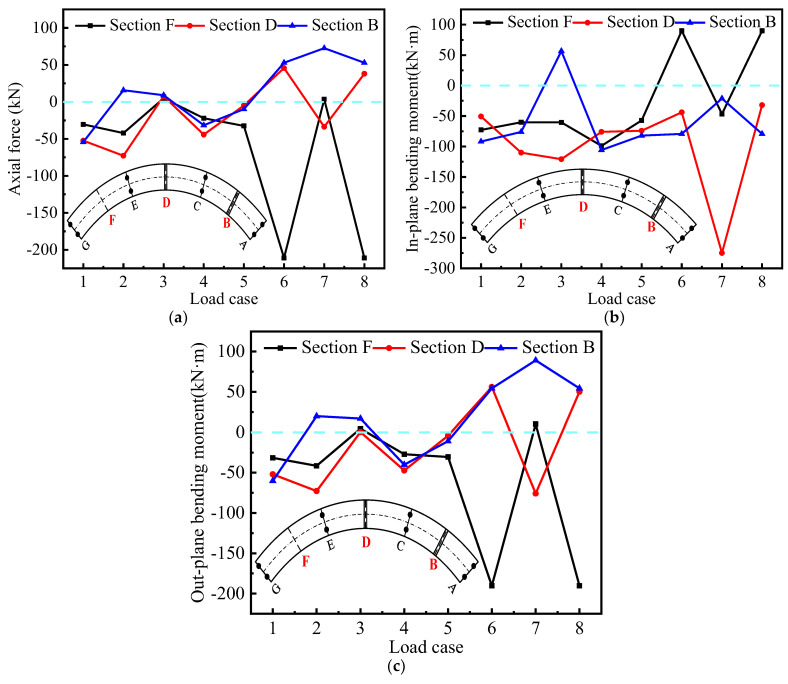
Investigation into box girder model internal forces with different loading cases: (**a**) sectional axial force; (**b**) sectional in-plane bending moment; (**c**) sectional in-plane bending moment. The A–G section represents the key sectionnumber of the bridge model.

Figure 18a shows the force submodel of axial force (N_j_), with different loading cases. The axial force in tension and compression of the control section also has a large difference, in general, limited by the number of loaded test blocks. The same loading case also shows the maximum axial force at the loaded section. Figure 18b shows the force submodel of the in-plane bending moment ($M_{j}^{in}$); here, the direction mainly shows each side of the top plate and bottom plate in tension and compression. Figure 18c shows the force submodel of out-of-plane bending moment ($M_{j}^{out}$), which can be used to represent the stressing state of the torsional moment, where the direction is mainly expressed as the force on the inside and outside of the box girder. As shown in Figure 18, by comparing the three different force sub-modal diagrams under the same loading case, it is confirmed which force sub-modal control each control section is subject to; for example, considering loading case 7, by comparative analysis, it is found that control section D is more susceptible to in-plane bending moment, while for loading cases 6 and 8, control section F is more susceptible to out-of-plane bending moment. This is because loading case 7 is symmetrically loaded while loading cases 6 and 8 are unilaterally loaded, which also indicates that torsional rollover is more likely to occur in this case.

The above situation shows that the axial internal force and out-of-plane bending moment mode can control the stressing state of the section with different loading cases, and the bending effect is still the main controlling factor of the box girder in general. Under symmetric loading, the out-of-plane torsional effect is not the main influencing factor of structural force, rather it is in-plane bending; under asymmetric loading cases, the out-of-plane torsional effect gradually becomes the main influencing factor of large curvature box girder force.

## 6. Conclusions

Eight different loading case stacking experiments of curved prestressed concrete box girder (CPCBG) bridges were conducted and *GSED* parameters and strain fields were constructed based on the structural stressing state theory and non-sample point interpolation method to reveal the working state and invisible mechanical properties of the CPCBG structures.

- The *GSED* obtained from the measured strain data is used to indicate the stressing state pattern of the structure based on the structural stressing state theory. The strain distribution mode of the bridge model, the various internal force values of the cross-section and the torsional effect under different loading cases are analyzed. It is found that the strain, deformation and force in the cross-section of the girder model with a partition beam are more coordinated than those of the bridge model without a partition beam.
- The torsional effect was constructed from the measured deflections, the out-of-plane pure torsional model from the rotational deflection and the in-plane bending model from the sinking deflection, both of which comprise the torsional effect of the curved beam, and the torsional effect maintained some coordination with the inboard and outboard strains. The presence of a partition beam affects the magnitude of internal forces such as axial forces; the overall stress level of the section with a partition beam is higher and more unevenly distributed than that of the section without a partition beam, and the torsional characteristics of the section with partition beam are different, but there is a significant improvement in the torsional effect of the section.
- Due to the presence of the torsional effect in the curved bridge model, the torsional stiffness is significantly smaller than the bending stiffness, and the torsional damage becomes the main factor. The curved bridge model also shows local damage under the action of a smaller stack load, which is more prominent in the inner side of the curved bridge model. The spacer beam has the function of maintaining the stability of the whole bridge in curved girder bridges. Especially for thin web box girders, adding spacers is the best way to reduce section deformation.
- An NPI method with clear physical meaning, based on reasonableness and accuracy, intuitively reflects the strain and stress fields. Torsional deformation easily leads to the outermost side of the bottom of the box girder and is the first to reach the cracking strain of the concrete. The damage to both sides of the box girder at local locations indicates that the structure is in an unstable stressing state, which intuitively reveals the torsional mechanical response of the three-box chamber box girder under the influence of a large curvature.
- The axial internal force and out-of-plane bending moment modes can reflect the force state of the cross-section, and, in general, the bending effect is still the main controlling factor of the box girder. In the symmetric loading case, in-plane bending is still the main influencing factor of structural forces, which has greater similarity with the general linear box girder forces; however, the out-of-plane torsional effect gradually becomes the main influencing factor of large curvature box girder forces under asymmetric loading.

The above study shows that the proposed NPI method and the torsional effect constructed by the structural stressing state theory can reveal the basic mechanical properties of the CPCBG bridge model. In conclusion, a clear perspective is adopted to analyze the mechanical properties of curved bridge models under different stacking conditions, which explores a new method for bridge engineering and provides a reference for engineering practice.

## Figures and Tables

**Table 1:** Concrete material mix ratio (kg/m^3^).

Water (kg)	Cement (kg)	Fly Coal Ash (kg)	Mineral Powder (kg)	Sand (kg)	Gravel (kg)	Pumping Agent (kg)
170	346	32	105	716	978	14

**Table 2:** Compressive strength of specimens at different ages.

Compressive Strength (MPa)	Bottom Plate Web/Top Plate			Bottom Plate Web/Top Plate		
Age(d)	7	14	28	7	14	28
Test sample 1	42.5	47.1	48.6	44.6	58.4	62.2
Test sample 2	39.9	47.6	50.5	41.3	57.1	55.0
Test sample 3	38.2	51.4	49.3	47.4	43.0	48.7
Mean	40.2	48.7	49.5	44.4	52.8	55.3
Compressive strength at 0.95 confidence interval	36.7	44.8	47.9	39.4	38.8	44.2

**Table 3:** Modulus of elasticity of test samples at the age of 28 days after standard curing.

Modulus of elasticity (GPa)	Sample 1	Sample 2	Sample 3	Mean
	35	29	35	33

**Table 4:** Mechanical properties of steel.

Material Type	Diameter (mm)	Ultimate Strength (MPa)	Yield Strength (MPa)	Elongation (%)	Modulus of Elasticity (GPa)
Prestressed steel strand	15.2	1978.4	1865.0	4.3	196.5
Ordinary steel bar	6	577.8	409.3	14.5	199.1

**Table 5:** Experimental loading case setting.

Loading Case	The Contents of the Loading Case	Loading Schematic Diagram
Case 1	Maximum bending moment at mid-span section F by live load	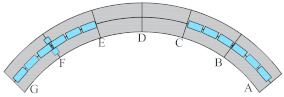
Case 2	Maximum bending moment at mid-span section B by live load	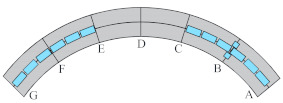
Case 3	Maximum bending moment at mid-span section D by live load	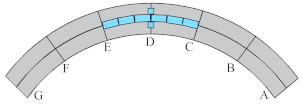
Case 4	Negative moment of support C by live load	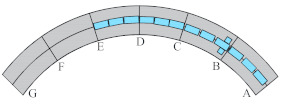
Case 5	Negative moment of support E by live load	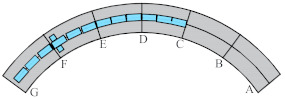
Case 6	The counterweight load for span E–G (Maximum positive bending moment in span F section)	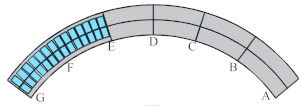
Case 7	The counterweight load for span C–E (Maximum positive bending moment in span D section)	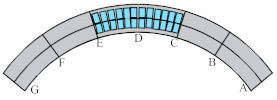
Case 8	The counterweight load for spans A–C (Maximum positive bending moment in span B section)	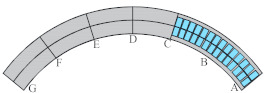

## Data Availability

Data is contained within the article.

## Appendix A

a. Experimental background: the unique bending-torsion coupling nature of bent girder bridges leads to their complex force characteristics, (manuscript table 294 lines). To further understand bent girder bridges, the manuscript focuses on a typical prestressed concrete bent box girder bridge with a flat curve radius of 12 m designed according to a scale of 1:6 as a prototype for a cross-river bridge project ramp quadruplex bridge as the engineering background for research and analysis.

b. Experimental setup: Shao (2017) [29] designed and tested a large curvature prestressed concrete three-span continuous curved box girder bridge model according to the scale of 1:6 and set eight load conditions (manuscript Table 5) to study the effects of the model bridge under constant load, live load, and prestressing, and tested the support reaction force, displacement and strain of the control section of the bridge under the above load conditions, respectively. They superimposed each constant load. The effects of the control section of the whole bridge under constant load are obtained by superimposing the conditions (manuscript table, line 386–388).

c. Experimental purpose: To study the force characteristics of small radius large span curved girder bridges through model tests of small radius large span curved girder bridges, to analyze their working characteristics with the test results, to further reveal the unseen bridge working characteristics, and, finally, to verify the validity of the model results calculated by the NPI method.

d. Equipment used in the test: The results to be measured in this test have a strain and displacement of the control section, the bearing reaction force. To achieve the measurement of the above physical quantities, resistive strain gauges, percentage displacement gauges, CL-YB-20T/2T resistive pressure ring, and a static strain acquisition system were used. Their quantities are shown in the following table.

**Table A1:** Summary of test equipment.

Instrument	Resistive Strain Gauges		Static Strain Gauge Kit			Displacement Gauges		Resistive Pressure Ring	
Machine type	BQ120-60AA	BE120-1AA	JM3813	JM3812	JM3815	YH-50	YH-1000L	CL-YB-20T	CL-YB-2T
Number	80	77	1	4	4	15	4	8	4

e. Principles of design use: Shao (2017) [29] designed and tested a large curvature prestressed concrete three-span continuous curved box girder bridge model according to a scale of 1:6, and the above tests satisfied the requirements of geometric similarity, boundary conditions, and physical conditions. All parts of the bridge (construction, mechanical calculation of prestressing tendon beam configuration, bearing configuration requirements, and structural durability design) meet the bridge design code requirements.

f. The loading conditions follow the procedures.

(1) The prestressing load was applied using unbonded prestressing strands, tensioned at both ends.
(2) Pre-loading was carried out before the test to bring all parts of the box girder model into working condition and coordinate with each other.
(3) The constant load of the bridge was applied by the counterweight, and the counterweight blocks were 26.5 cm × 48 cm × 92 cm steel plate concrete blocks, each weighing 300 kg. Due to a large number of counterweights, single-span loading was used here, and the effects were superimposed at the end, which reduced the number of counterweights and reduced the difficulty of the test, and also ensured the smooth conduct of the test.
(4) The live load is also loaded utilizing counterweight blocks, and the loading quantity is calculated according to the internal force effect of each control section under the design load, and the load is arranged according to the principle of influence line to achieve the most unfavorable effect of the control section. The loading sequence of counterweight blocks is from the inside to the outside and from the support to the middle of the span. The loading process is continuous and does not allow too long a pause in the middle process.

g. The way of collecting data and other information.

The measured data are strain and displacement of the control section, bearing reaction force and strain gauges of the corresponding measurement points are pasted on the surface of the beam, displacement gauges are arranged to the key position, and pressure transducers are arranged under the bearing to measure the bearing reaction force. To realize the measurement of the above physical quantities, instruments such as resistive strain gauges, electronic displacement gauges, and resistive pressure rings are used, and the data at the output are connected to a static wired collection system for collection (the JM3813 static strain acquisition box) with a data sampling frequency of 200 Hz (T = 0.005 s).

h. What inference or effect is obtained.

(a) The test main beam shows a torsional effect, which is mainly manifested as follows: transverse pattern of bearing and span displacement: large outer displacement, small inner displacement; the transverse pattern of bearing reaction force: large outer bearing reaction force, small inner bearing reaction force.

i. How to further analyze the data.

The following types of data are mainly obtained through the test: bearing reaction force, the strain of web and top plate of branch section, the strain of web and top plate of span section, displacement of bearing and span section.

(1) Bearing reaction force is used for the transverse law of bearing reaction force.
(2) The strains of each key section are used for the study of the sum of *GSED* (*E_sum_*), stressing state distribution and NPI interpolation calculation, etc.
(3) The displacements of each key section are used to construct the torsional effect.

## Appendix B

**Table A2:** Sum of generalized strain energy density *GSED* for each section with different loading cases (*E_j_*).

*E_j_* (J/m^3^)		Loading Case							
		Case 1	Case 2	Case 3	Case 4	Case 5	Case 6	Case 7	Case 8
Key cross sections	B	1470.2	2784.1	2451.3	2839	2990.2	35.7	122.7	35.7
	C	45	52.6	11.1	6.5	6.6	13.1	351.2	9.7
	D	61.1	63.2	2580.5	175.2	349.2	82.6	1966.7	88.4
	E	6.1	19.6	2.2	7.4	8.3	155.6	305.7	155.6
	F	52.7	45	3.5	49	36.4	1400.2	110.5	1400.2
